# Supplementary material for: Conspecific and interspecific stimuli reduce initial performance in an aversive learning task in honey bees (Apis mellifera)
Source: PLoS One. 2020 Feb 25;15(2):e0228161. doi: 10.1371/journal.pone.0228161 (PMC7041878; doi:10.1371/journal.pone.0228161)
Supplement: S1 Table — (DOCX) [file pone.0228161.s001.docx]

Table S1

| *Experimental Groups CRR Analysis – Group Effects* | | | |
| --- | --- | --- | --- |
| Pairwise Comparison | Difference | Standard Error | *p*-value |
| Spatial : Safe by live bee | 11.161 | 5.594 | 0.046 |
| Spatial : Shock by live bee | 1.982 | 5.757 | 0.731 |
| Spatial : Safe by dead bee | 10.849 | 6.224 | 0.081 |
| Spatial : Shock by dead bee | 10.345 | 7.021 | 0.141 |
| Spatial : Safe by live wasp | 4.689 | 6.277 | 0.455 |
| Spatial : Shock by live wasp | 5.781 | 6.410 | 0.367 |
| Spatial : Safe by dead wasp | 10.655 | 5.671 | 0.060 |
| Spatial : Shock by dead wasp | 9.727 | 6.091 | 0.110 |
| Safe by live bee : Shock by live bee | -9.179 | 5.191 | 0.077 |
| Safe by live bee : Safe by dead bee | -0.313 | 5.705 | 0.956 |
| Safe by live bee : Shock by dead bee | -0.816 | 6.565 | 0.901 |
| Safe by live bee : Safe by live wasp | -6.473 | 5.763 | 0.261 |
| Safe by live bee : Shock by live wasp | -5.380 | 5.907 | 0.362 |
| Safe by live bee : Safe by dead wasp | -0.506 | 5.096 | 0.921 |
| Safe by live bee : Shock by dead wasp | -1.435 | 5.559 | 0.796 |
| Shock by live bee : Safe by dead bee | 8.866 | 5.864 | 0.131 |
| Shock by live bee : Shock by dead bee | 8.363 | 6.704 | 0.212 |
| Shock by live bee : Safe by live wasp | 2.706 | 5.920 | 0.648 |
| Shock by live bee : Shock by live wasp | 3.799 | 6.061 | 0.531 |
| Shock by live bee : Safe by dead wasp | 8.673 | 5.274 | 0.100 |
| Shock by live bee : Shock by dead wasp | 7.744 | 5.723 | 0.176 |
| Safe by dead bee : Shock by dead bee | -0.503 | 7.109 | 0.944 |
| Safe by dead bee : Safe by live wasp | -6.160 | 6.376 | 0.334 |
| Safe by dead bee : Shock by live wasp | -5.067 | 6.506 | 0.436 |
| Safe by dead bee : Safe by dead wasp | -0.193 | 5.780 | 0.973 |
| Safe by dead bee : Shock by dead wasp | -1.122 | 6.193 | 0.856 |
| Shock by dead bee : Safe by live wasp | -5.657 | 7.155 | 0.429 |
| Shock by dead bee : Shock by live wasp | -4.564 | 7.272 | 0.530 |
| Shock by dead bee : Safe by dead wasp | 0.310 | 6.630 | 0.963 |
| Shock by dead bee : Shock by dead wasp | -0.618 | 6.993 | 0.930 |
| Safe by live wasp : Shock by live wasp | 1.093 | 6.557 | 0.868 |
| Safe by live wasp : Safe by dead wasp | 5.967 | 5.837 | 0.307 |
| Safe by live wasp : Shock by dead wasp | 5.038 | 6.246 | 0.420 |
| Shock by live wasp : Safe by dead wasp | 4.874 | 5.979 | 0.415 |
| Shock by live wasp : Shock by dead wasp | 3.945 | 6.379 | 0.536 |
| Safe by dead wasp : Shock by dead wasp | -0.929 | 5.637 | 0.869 |

\
