# Supplementary material for: Conspecific and interspecific stimuli reduce initial performance in an aversive learning task in honey bees (Apis mellifera)
Source: PLoS One. 2020 Feb 25;15(2):e0228161. doi: 10.1371/journal.pone.0228161 (PMC7041878; doi:10.1371/journal.pone.0228161)
Supplement: S3 Table — (DOCX) [file pone.0228161.s003.docx]

Table S3

| *Experimental Groups Activity Analysis – Group Effects* | | | |
| --- | --- | --- | --- |
| Pairwise Comparison | Difference | Standard Error | *p*-value |
| Spatial : Safe by live bee | -0.318 | 0.234 | 0.175 |
| Spatial : Shock by live bee | -0.185 | 0.220 | 0.399 |
| Spatial : Safe by dead bee | -0.418 | 0.287 | 0.145 |
| Spatial : Shock by dead bee | -0.439 | 0.323 | 0.174 |
| Spatial : Safe by live wasp | -0.587 | 0.314 | 0.062 |
| Spatial : Shock by live wasp | -0.030 | 0.239 | 0.899 |
| Spatial : Safe by dead wasp | -0.433 | 0.246 | 0.079 |
| Spatial : Shock by dead wasp | -0.353 | 0.283 | 0.213 |
| Safe by live bee : Shock by live bee | 0.132 | 0.215 | 0.539 |
| Safe by live bee : Safe by dead bee | -0.100 | 0.283 | 0.724 |
| Safe by live bee : Shock by dead bee | -0.121 | 0.320 | 0.704 |
| Safe by live bee : Safe by live wasp | -0.270 | 0.311 | 0.387 |
| Safe by live bee : Shock by live wasp | 0.287 | 0.235 | 0.222 |
| Safe by live bee : Safe by dead wasp | -0.115 | 0.242 | 0.635 |
| Safe by live bee : Shock by dead wasp | -0.035 | 0.280 | 0.900 |
| Shock by live bee : Safe by dead bee | -0.232 | 0.272 | 0.392 |
| Shock by live bee : Shock by dead bee | -0.254 | 0.310 | 0.412 |
| Shock by live bee : Safe by live wasp | -0.402 | 0.301 | 0.181 |
| Shock by live bee : Shock by live wasp | 0.155 | 0.221 | 0.483 |
| Shock by live bee : Safe by dead wasp | -0.247 | 0.228 | 0.278 |
| Shock by live bee : Shock by dead wasp | -0.168 | 0.268 | 0.532 |
| Safe by dead bee : Shock by dead bee | -0.021 | 0.360 | 0.953 |
| Safe by dead bee : Safe by live wasp | -0.169 | 0.353 | 0.631 |
| Safe by dead bee : Shock by live wasp | 0.387 | 0.288 | 0.178 |
| Safe by dead bee : Safe by dead wasp | -0.015 | 0.293 | 0.959 |
| Safe by dead bee : Shock by dead wasp | 0.065 | 0.325 | 0.842 |
| Shock by dead bee : Safe by live wasp | -0.148 | 0.383 | 0.699 |
| Shock by dead bee : Shock by live wasp | 0.409 | 0.324 | 0.207 |
| Shock by dead bee : Safe by dead wasp | 0.006 | 0.329 | 0.985 |
| Shock by dead bee : Shock by dead wasp | 0.086 | 0.358 | 0.810 |
| Safe by live wasp : Shock by live wasp | 0.557 | 0.315 | 0.077 |
| Safe by live wasp : Safe by dead wasp | 0.154 | 0.320 | 0.630 |
| Safe by live wasp : Shock by dead wasp | 0.234 | 0.350 | 0.503 |
| Shock by live wasp : Safe by dead wasp | -0.402 | 0.247 | 0.103 |
| Shock by live wasp : Shock by dead wasp | -0.323 | 0.284 | 0.256 |
| Safe by dead wasp : Shock by dead wasp | 0.080 | 0.290 | 0.783 |
