# Supplementary material for: Conspecific and interspecific stimuli reduce initial performance in an aversive learning task in honey bees (Apis mellifera)
Source: PLoS One. 2020 Feb 25;15(2):e0228161. doi: 10.1371/journal.pone.0228161 (PMC7041878; doi:10.1371/journal.pone.0228161)
Supplement: S4 Table — (DOCX) [file pone.0228161.s004.docx]

Table S4

| *Experimental Activity CRR Analysis – Bin Interactions* | | | |
| --- | --- | --- | --- |
| Pairwise Comparison | Difference | Standard Error | *p*-value |
| Spatial X Bin : Safe by live bee X Bin | 0.003 | 0.015 | 0.860 |
| Spatial X Bin : Shock by live bee X Bin | -0.008 | 0.013 | 0.557 |
| Spatial X Bin : Safe by dead bee X Bin | 0.004 | 0.018 | 0.829 |
| Spatial X Bin : Shock by dead bee X Bin | 0.013 | 0.021 | 0.535 |
| Spatial X Bin : Safe by live wasp X Bin | -0.001 | 0.018 | 0.965 |
| Spatial X Bin : Shock by live wasp X Bin | -0.017 | 0.015 | 0.245 |
| Spatial X Bin : Safe by dead wasp X Bin | 0.010 | 0.017 | 0.546 |
| Spatial X Bin : Shock by dead wasp X Bin | -0.015 | 0.018 | 0.409 |
| Safe by live bee X Bin : Shock by live bee X Bin | -0.010 | 0.016 | 0.504 |
| Safe by live bee X Bin : Safe by dead bee X Bin | 0.001 | 0.020 | 0.949 |
| Safe by live bee X Bin : Shock by dead bee X Bin | 0.010 | 0.022 | 0.650 |
| Safe by live bee X Bin : Safe by live wasp X Bin | -0.003 | 0.020 | 0.861 |
| Safe by live bee X Bin : Shock by live wasp X Bin | -0.020 | 0.017 | 0.242 |
| Safe by live bee X Bin : Safe by dead wasp X Bin | 0.007 | 0.019 | 0.695 |
| Safe by live bee X Bin : Shock by dead wasp X Bin | -0.018 | 0.020 | 0.377 |
| Shock by live bee X Bin : Safe by dead bee X Bin | 0.012 | 0.019 | 0.531 |
| Shock by live bee X Bin : Shock by dead bee X Bin | 0.021 | 0.021 | 0.326 |
| Shock by live bee X Bin : Safe by live wasp X Bin | 0.007 | 0.018 | 0.705 |
| Shock by live bee X Bin : Shock by live wasp X Bin | -0.009 | 0.015 | 0.532 |
| Shock by live bee X Bin : Safe by dead wasp X Bin | 0.018 | 0.017 | 0.292 |
| Shock by live bee X Bin : Shock by dead wasp X Bin | -0.007 | 0.018 | 0.699 |
| Safe by dead bee X Bin : Shock by dead bee X Bin | 0.009 | 0.025 | 0.719 |
| Safe by dead bee X Bin : Safe by live wasp X Bin | -0.005 | 0.023 | 0.831 |
| Safe by dead bee X Bin : Shock by live wasp X Bin | -0.021 | 0.020 | 0.286 |
| Safe by dead bee X Bin : Safe by dead wasp X Bin | 0.006 | 0.021 | 0.778 |
| Safe by dead bee X Bin : Shock by dead wasp X Bin | -0.019 | 0.022 | 0.400 |
| Shock by dead bee X Bin : Safe by live wasp X Bin | -0.014 | 0.024 | 0.576 |
| Shock by dead bee X Bin : Shock by live wasp X Bin | -0.030 | 0.022 | 0.172 |
| Shock by dead bee X Bin : Safe by dead wasp X Bin | -0.003 | 0.023 | 0.901 |
| Shock by dead bee X Bin : Shock by dead wasp X Bin | -0.028 | 0.024 | 0.254 |
| Safe by live wasp X Bin : Shock by live wasp X Bin | -0.016 | 0.020 | 0.401 |
| Safe by live wasp X Bin : Safe by dead wasp X Bin | 0.011 | 0.021 | 0.607 |
| Safe by live wasp X Bin : Shock by dead wasp X Bin | -0.014 | 0.022 | 0.525 |
| Shock by live wasp X Bin : Safe by dead wasp X Bin | 0.027 | 0.018 | 0.134 |
| Shock by live wasp X Bin : Shock by dead wasp X Bin | 0.002 | 0.019 | 0.900 |
| Safe by dead wasp X Bin : Shock by dead wasp X Bin | -0.025 | 0.021 | 0.233 |
