# Supplementary material for: Conspecific and interspecific stimuli reduce initial performance in an aversive learning task in honey bees (Apis mellifera)
Source: PLoS One. 2020 Feb 25;15(2):e0228161. doi: 10.1371/journal.pone.0228161 (PMC7041878; doi:10.1371/journal.pone.0228161)
Supplement: S6 Table — (DOCX) [file pone.0228161.s006.docx]

Table S6

| *Experimental Groups CRR Analysis – Bin Interactions* | | | |
| --- | --- | --- | --- |
| Pairwise Comparison | Difference | Standard Error | *p*-value |
| Spatial X Bin : Safe by live bee X Bin | -0.202 | 0.443 | 0.649 |
| Spatial X Bin : Shock by live bee X Bin | 0.281 | 0.477 | 0.557 |
| Spatial X Bin : Safe by dead bee X Bin | -0.274 | 0.484 | 0.572 |
| Spatial X Bin : Shock by dead bee X Bin | -0.408 | 0.529 | 0.441 |
| Spatial X Bin : Safe by live wasp X Bin | 0.530 | 0.435 | 0.223 |
| Spatial X Bin : Shock by live wasp X Bin | -0.066 | 0.457 | 0.885 |
| Spatial X Bin : Safe by dead wasp X Bin | -0.230 | 0.387 | 0.553 |
| Spatial X Bin : Shock by dead wasp X Bin | 0.004 | 0.518 | 0.994 |
| Safe by live bee X Bin : Shock by live bee X Bin | 0.483 | 0.487 | 0.322 |
| Safe by live bee X Bin : Safe by dead bee X Bin | -0.072 | 0.494 | 0.884 |
| Safe by live bee X Bin : Shock by dead bee X Bin | -0.206 | 0.538 | 0.702 |
| Safe by live bee X Bin : Safe by live wasp X Bin | 0.732 | 0.445 | 0.100 |
| Safe by live bee X Bin : Shock by live wasp X Bin | 0.136 | 0.467 | 0.771 |
| Safe by live bee X Bin : Safe by dead wasp X Bin | -0.028 | 0.399 | 0.945 |
| Safe by live bee X Bin : Shock by dead wasp X Bin | 0.206 | 0.527 | 0.696 |
| Shock by live bee X Bin : Safe by dead bee X Bin | -0.554 | 0.525 | 0.291 |
| Shock by live bee X Bin : Shock by dead bee X Bin | -0.688 | 0.566 | 0.224 |
| Shock by live bee X Bin : Safe by live wasp X Bin | 0.249 | 0.479 | 0.603 |
| Shock by live bee X Bin : Shock by live wasp X Bin | -0.347 | 0.500 | 0.488 |
| Shock by live bee X Bin : Safe by dead wasp X Bin | -0.510 | 0.437 | 0.243 |
| Shock by live bee X Bin : Shock by dead wasp X Bin | -0.277 | 0.556 | 0.618 |
| Safe by dead bee X Bin : Shock by dead bee X Bin | -0.134 | 0.572 | 0.815 |
| Safe by dead bee X Bin : Safe by live wasp X Bin | 0.804 | 0.486 | 0.098 |
| Safe by dead bee X Bin : Shock by live wasp X Bin | 0.208 | 0.506 | 0.681 |
| Safe by dead bee X Bin : Safe by dead wasp X Bin | 0.044 | 0.444 | 0.921 |
| Safe by dead bee X Bin : Shock by dead wasp X Bin | 0.278 | 0.562 | 0.621 |
| Shock by dead bee X Bin : Safe by live wasp X Bin | 0.938 | 0.530 | 0.077 |
| Shock by dead bee X Bin : Shock by live wasp X Bin | 0.342 | 0.549 | 0.534 |
| Shock by dead bee X Bin : Safe by dead wasp X Bin | 0.178 | 0.492 | 0.718 |
| Shock by dead bee X Bin : Shock by dead wasp X Bin | 0.412 | 0.600 | 0.493 |
| Safe by live wasp X Bin : Shock by live wasp X Bin | -0.596 | 0.459 | 0.194 |
| Safe by live wasp X Bin : Safe by dead wasp X Bin | -0.760 | 0.389 | 0.051 |
| Safe by live wasp X Bin : Shock by dead wasp X Bin | -0.526 | 0.519 | 0.311 |
| Shock by live wasp X Bin : Safe by dead wasp X Bin | -0.164 | 0.414 | 0.693 |
| Shock by live wasp X Bin : Shock by dead wasp X Bin | 0.070 | 0.538 | 0.897 |
| Safe by dead wasp X Bin : Shock by dead wasp X Bin | 0.233 | 0.480 | 0.627 |
