# Supplementary figures and images for: Conspecific and interspecific stimuli reduce initial performance in an aversive learning task in honey bees (Apis mellifera)
Source: PLoS One. 2020 Feb 25;15(2):e0228161. doi: 10.1371/journal.pone.0228161 (PMC7041878; doi:10.1371/journal.pone.0228161)

**
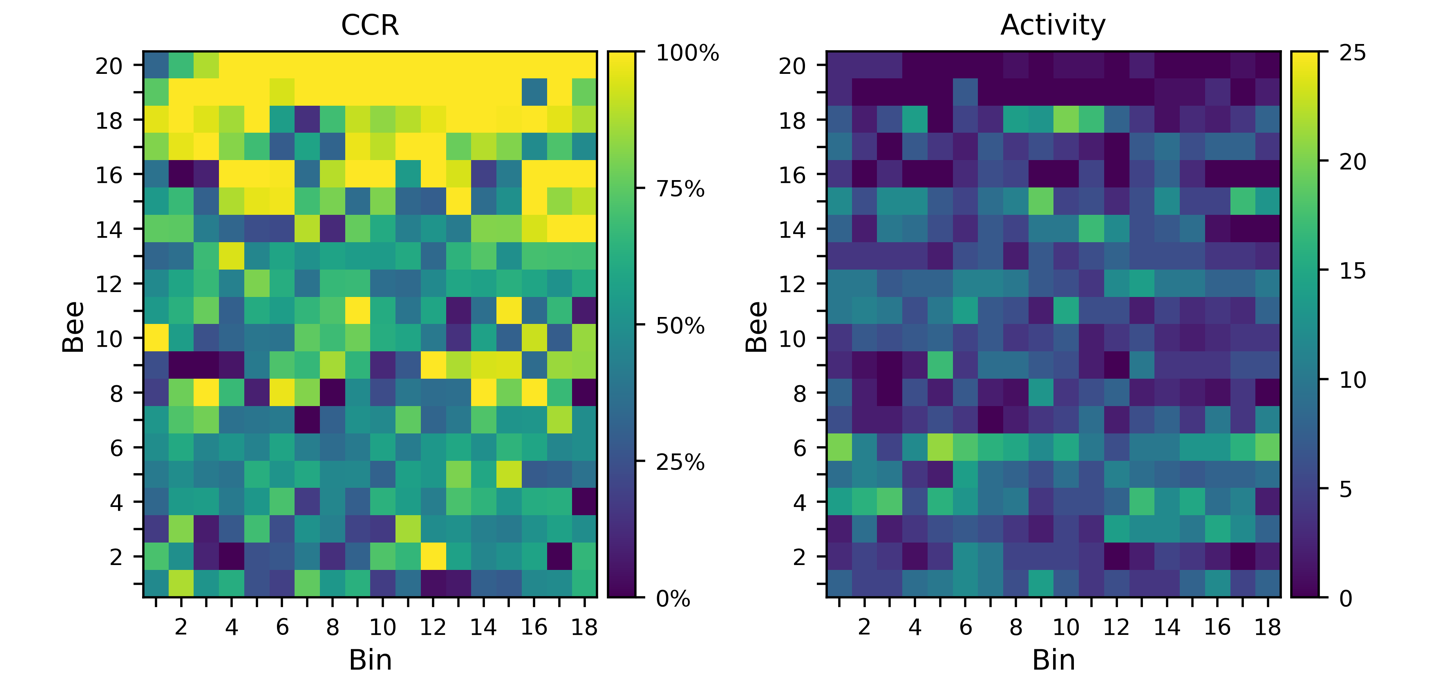
Figure S1.** Heat map of CCR and activity levels of the spatial group.

Supplement: S1 Fig — (DOCX) [file pone.0228161.s009.docx]

**
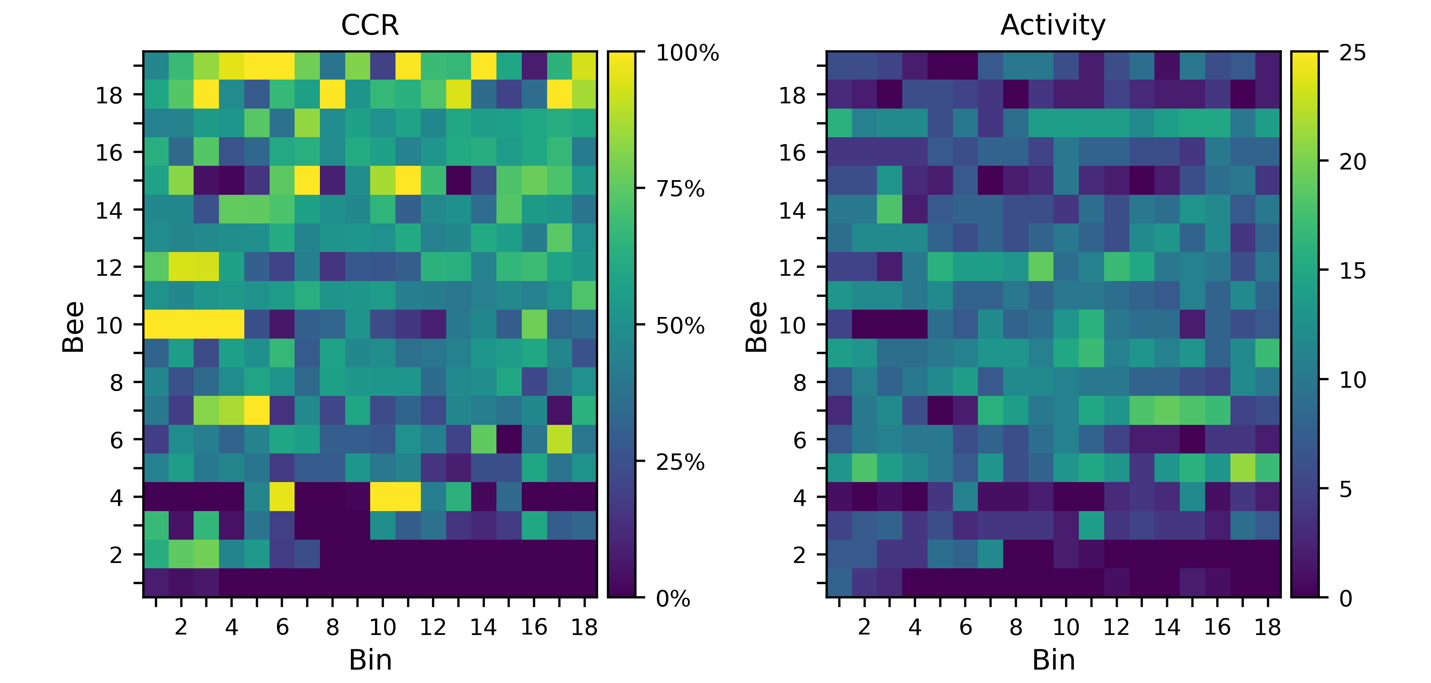
Figure S2.** Heat map of CCR and activity levels of the shock on blue group.

Supplement: S2 Fig — (DOCX) [file pone.0228161.s010.docx]

**
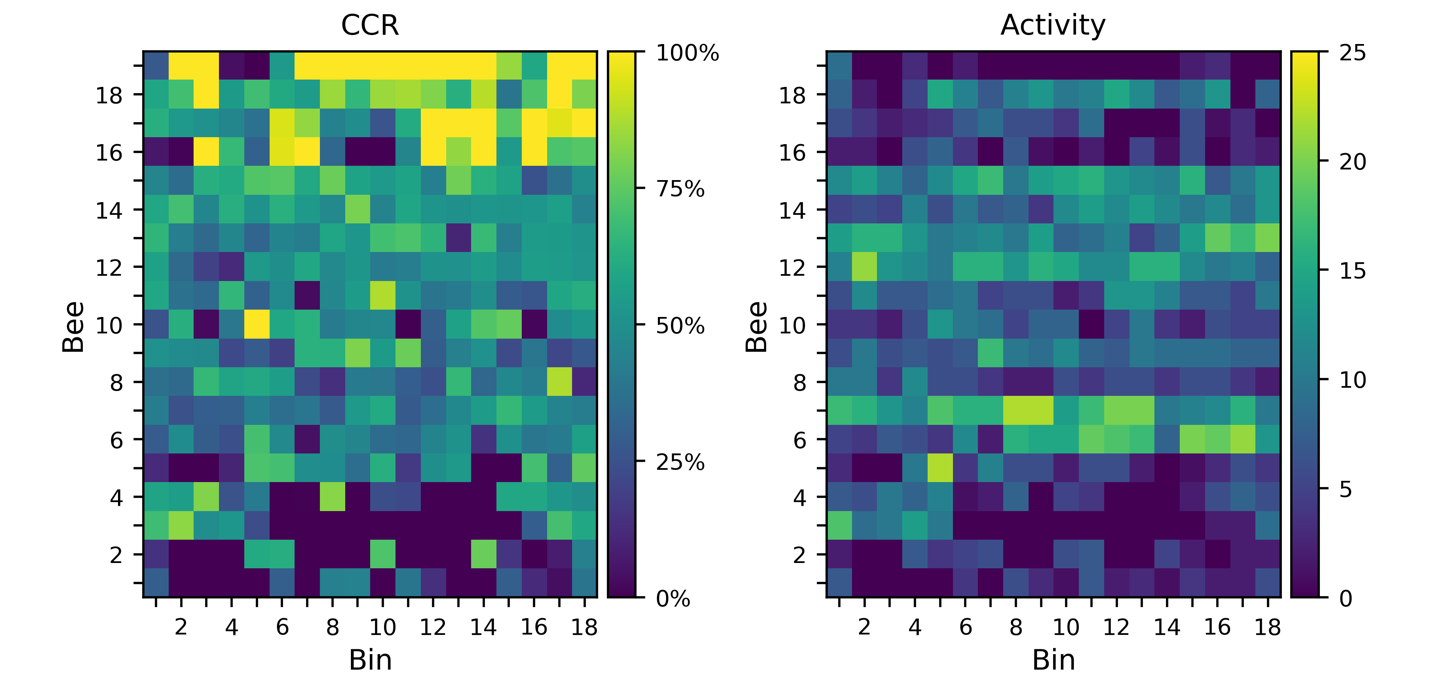
Figure S3.** Heat map of CCR and activity levels of the shock on yellow group.

Supplement: S3 Fig — (DOCX) [file pone.0228161.s011.docx]

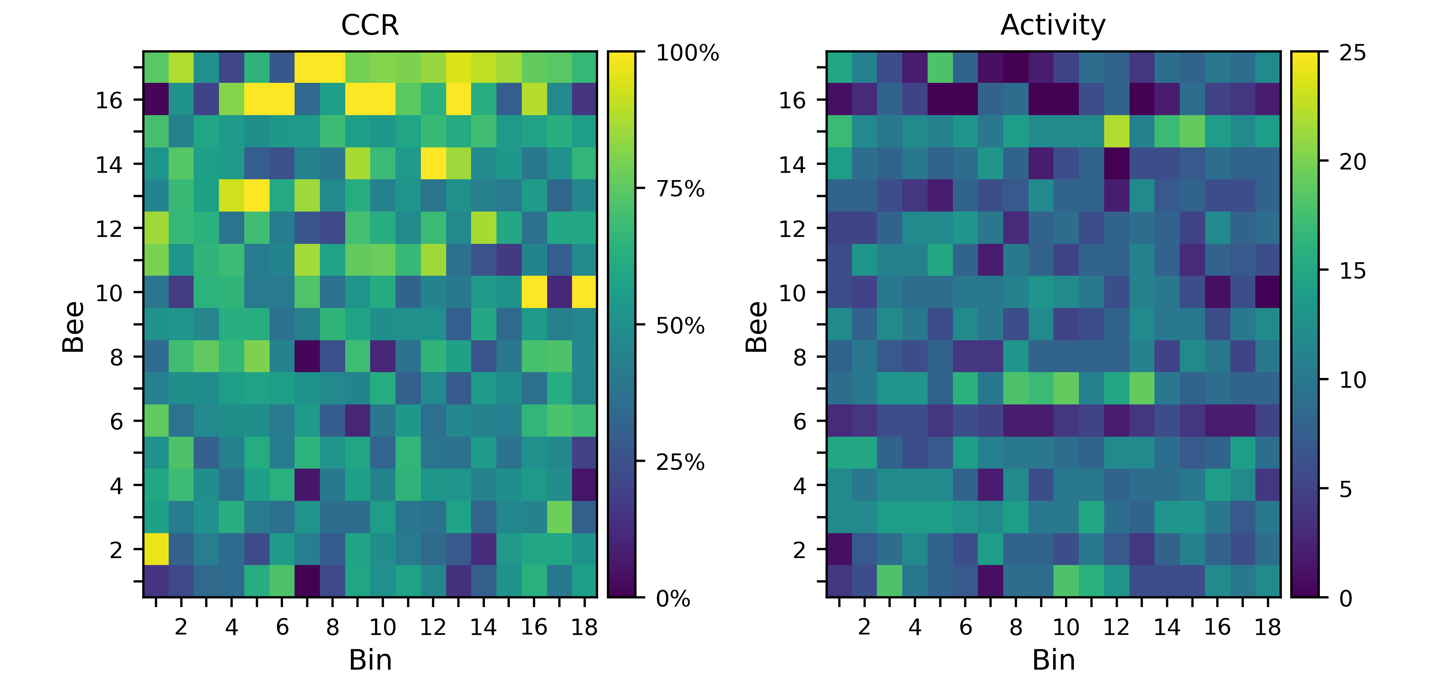


**Figure S4.** Heat map of CCR and activity levels of the bee baseline group.

Supplement: S4 Fig — (DOCX) [file pone.0228161.s012.docx]

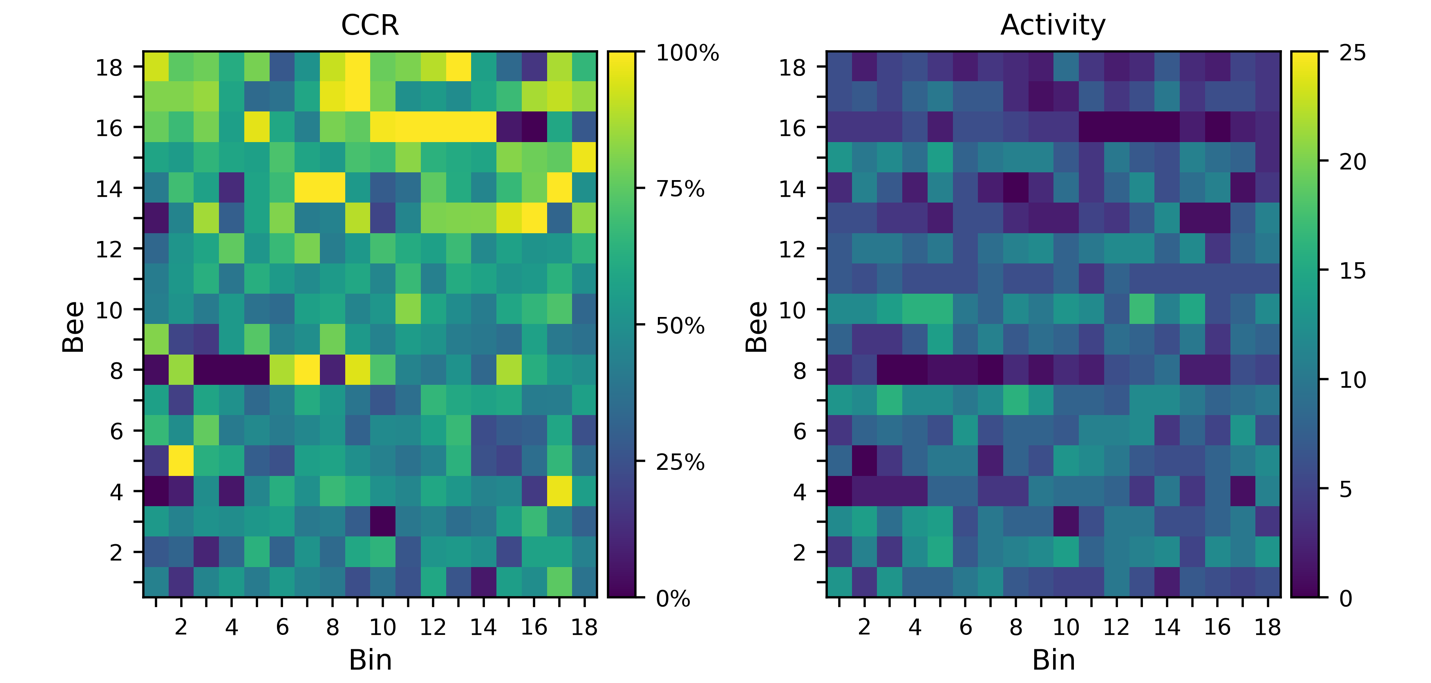


**Figure S5.** Heat map of CCR and activity levels of the wasp baseline group.

Supplement: S5 Fig — (DOCX) [file pone.0228161.s013.docx]

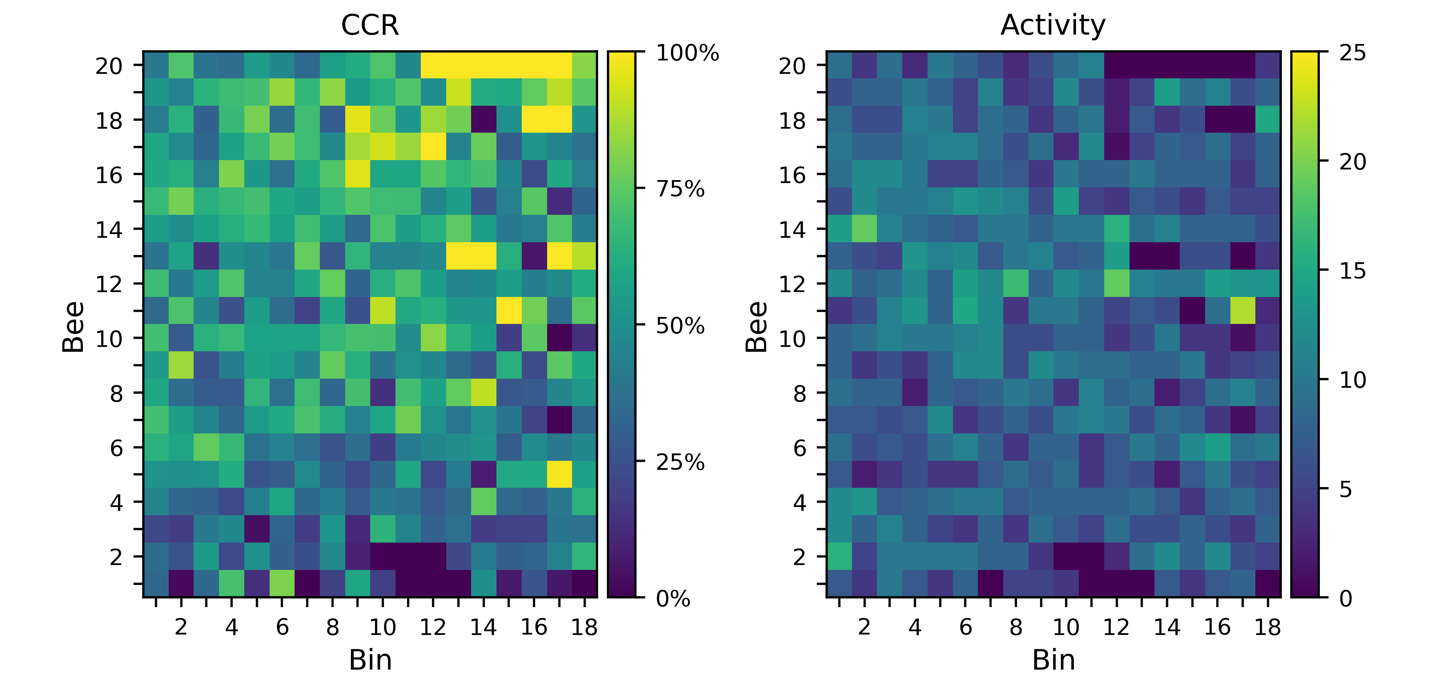


**Figure S6.** Heat map of CCR and activity levels of the bee and wasp baseline group.

Supplement: S6 Fig — (DOCX) [file pone.0228161.s014.docx]

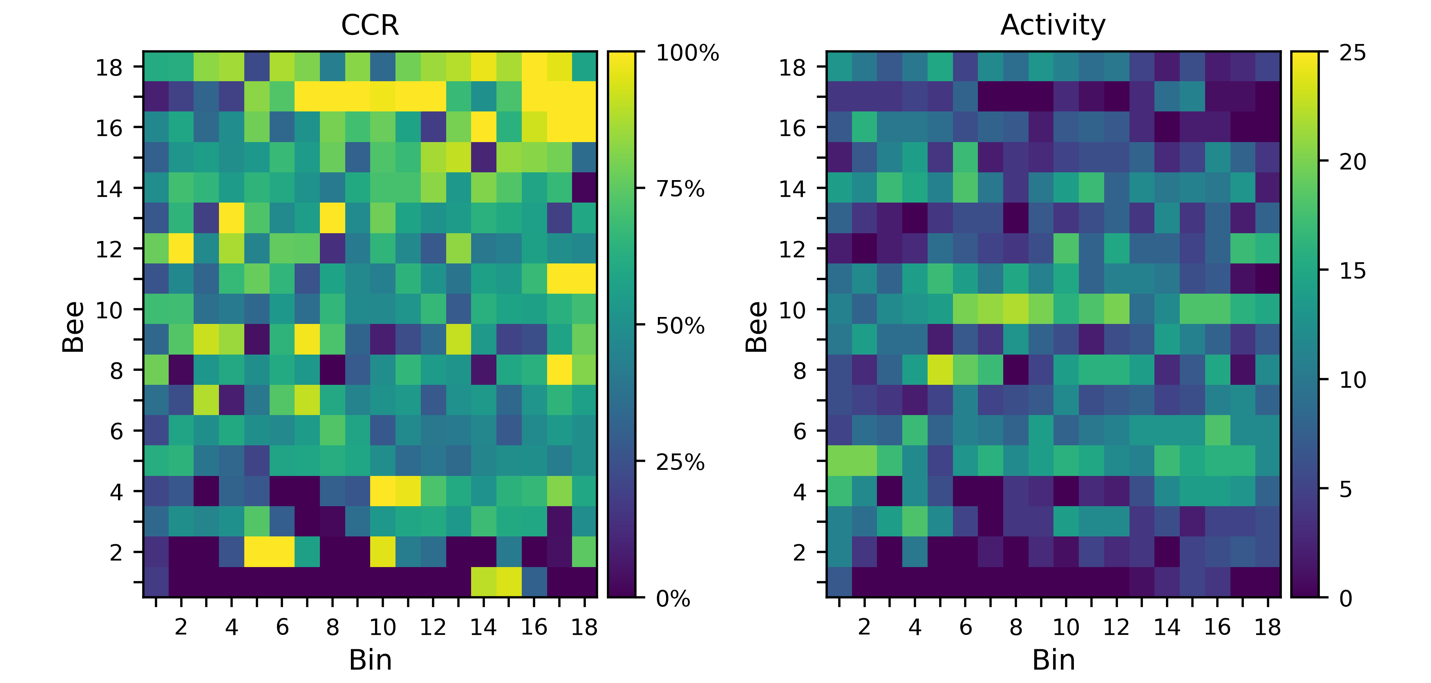


**Figure S9.** Heat map of CCR and activity levels of the safe by dead bee group.

Supplement: S9 Fig — (DOCX) [file pone.0228161.s017.docx]

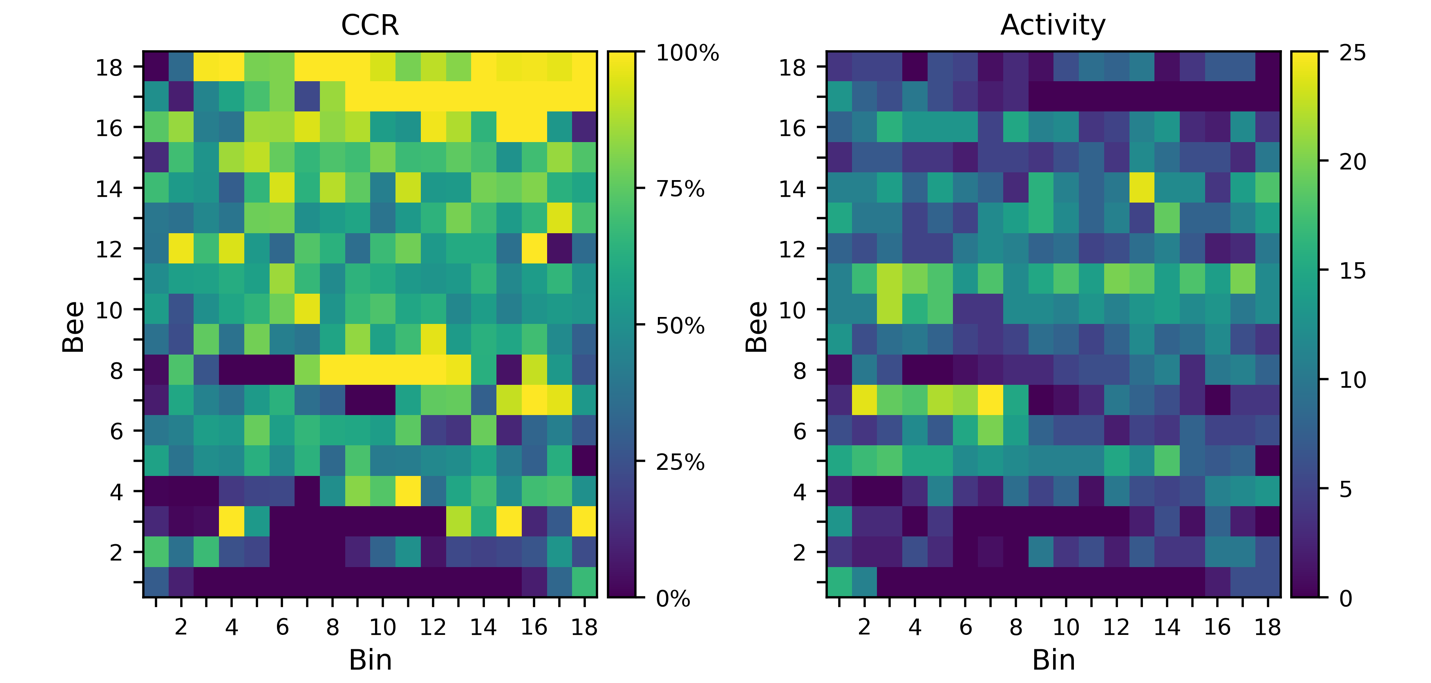


**Figure S10.** Heat map of CCR and activity levels of the shock by dead bee group.

Supplement: S10 Fig — (DOCX) [file pone.0228161.s018.docx]

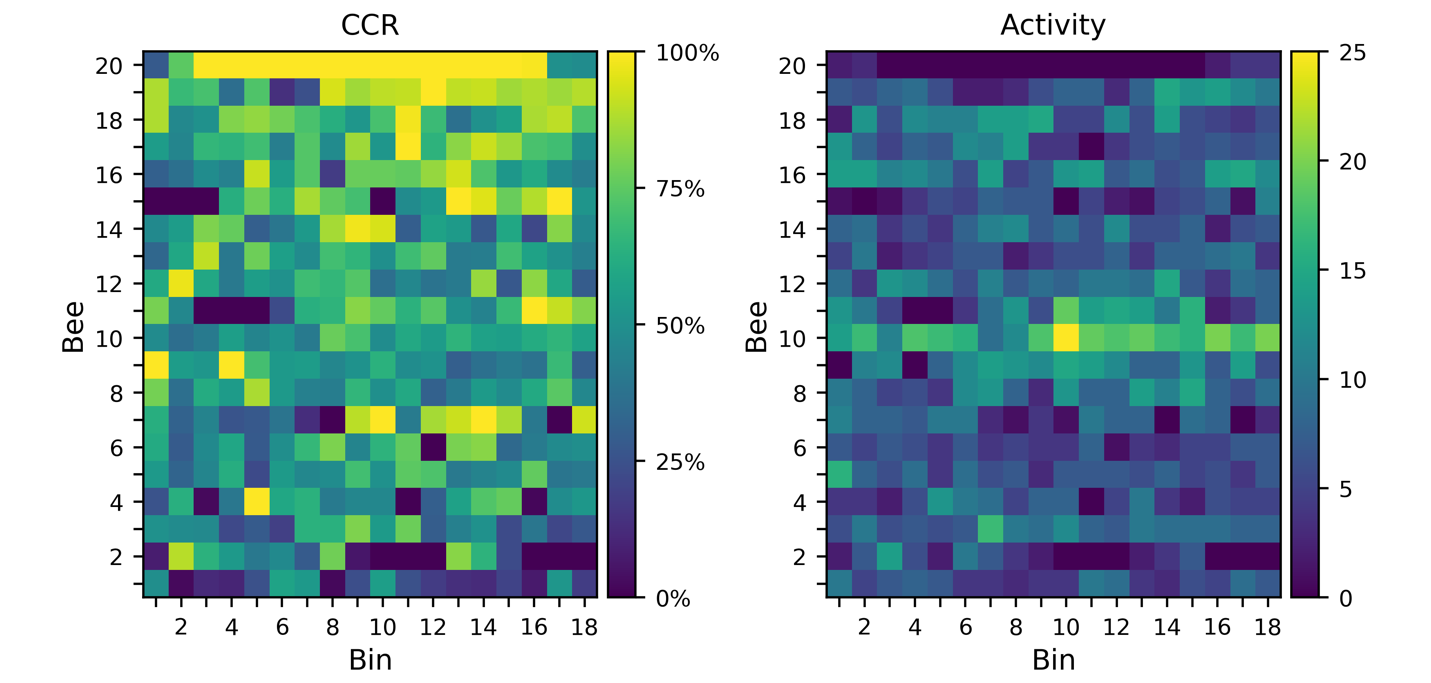


**Figure S12.** Heat map of CCR and activity levels of the shock by live wasp group.

Supplement: S12 Fig — (DOCX) [file pone.0228161.s020.docx]

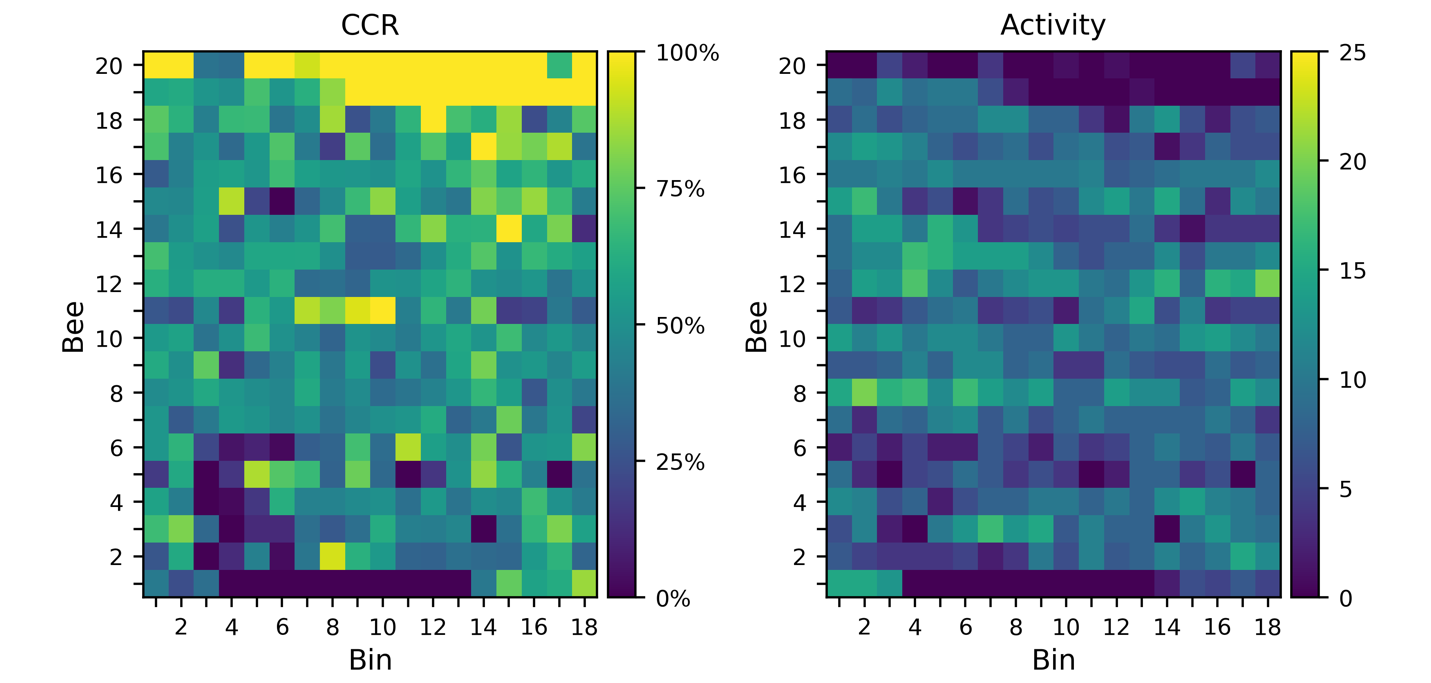


**Figure S13.** Heat map of CCR and activity levels of the safe by dead wasp group.

Supplement: S13 Fig — (DOCX) [file pone.0228161.s021.docx]
