# Supplementary material for: Conspecific and interspecific stimuli reduce initial performance in an aversive learning task in honey bees (Apis mellifera)
Source: PLoS One. 2020 Feb 25;15(2):e0228161. doi: 10.1371/journal.pone.0228161 (PMC7041878; doi:10.1371/journal.pone.0228161)
Supplement: S8 Fig — Note that one extreme activity score was truncated to 25 for sake of consistency across graphs. (DOCX) [file pone.0228161.s016.docx]

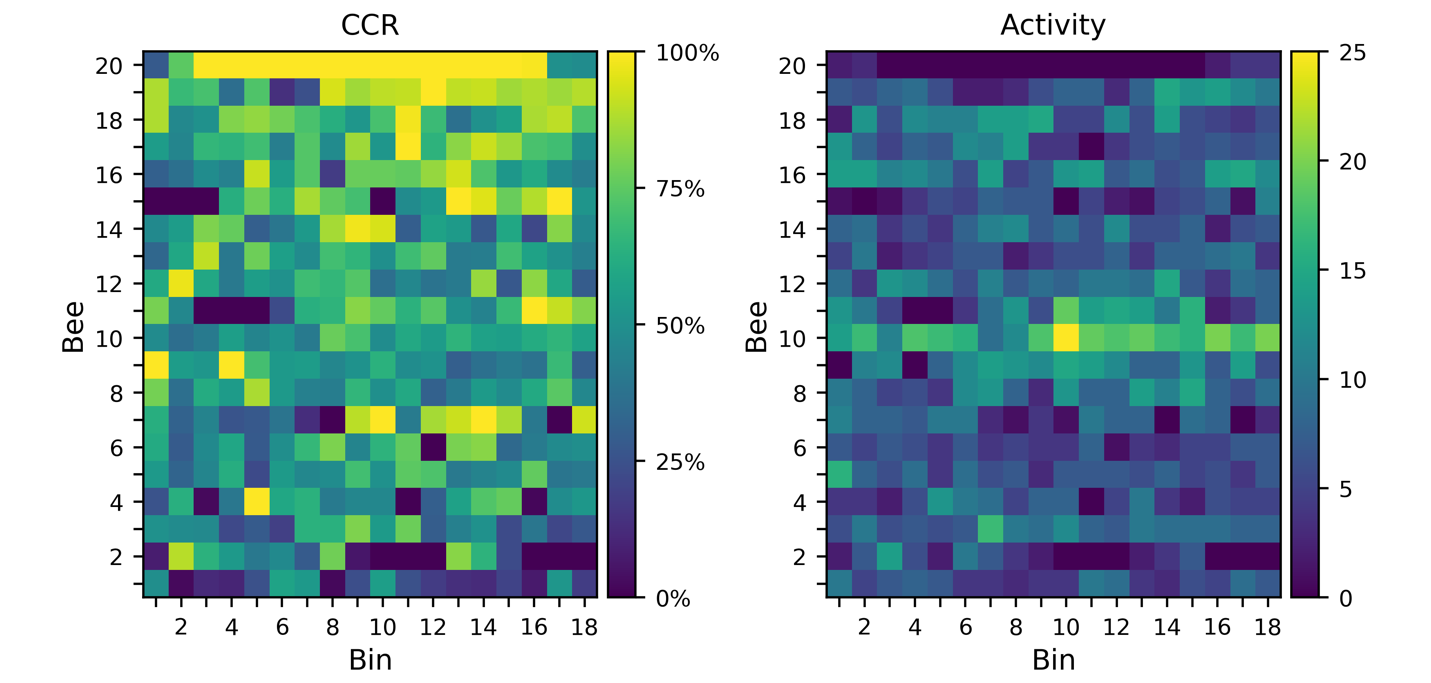


**Figure S8.** Heat map of CCR and activity levels of the shock by live bee group. Note that one extreme activity score was truncated to 25 for sake of consistency across graphs.
