# Supplementary material for: Conspecific and interspecific stimuli reduce initial performance in an aversive learning task in honey bees (Apis mellifera)
Source: PLoS One. 2020 Feb 25;15(2):e0228161. doi: 10.1371/journal.pone.0228161 (PMC7041878; doi:10.1371/journal.pone.0228161)
Supplement: Attachment — Submitted filename: Response to Reviewers.docx [file pone.0228161.s025.docx]

**Response to Editor and Reviewer Comments**

*We are grateful for the insightful comments of the reviewers (see our acknowledgements). We have substantially revised our manuscript based on the comments of the editor and reviewers. We address specific comments below, in italics.*

**General Formatting Comments**

In your Methods section, please provide additional location information of the collections site, including geographic coordinates for the data set if available.

*We included latitude and longitude coordinates for Stillwater, Oklahoma.*

In your Methods section, please provide additional information regarding the permits you obtained for the work. Please ensure you have included the full name of the authority that approved the collection site access and, if no permits were required, a brief statement explaining why.

*Our work follows Oklahoma State University regulations. No permits or IACUC approval were required for this research. We initially included the following two statements in the manuscript, and we believe these statements meet the requirements of PLOS ONE.*

*As Oklahoma State University does not require an ethics institutional review for non-threatened invertebrates, no specific review or permits were required to collect subjects for the present study.*

*As Oklahoma State University does not require an ethics institutional review for non-threatened invertebrates, no specific review or permits were required to collect stimulus animals for the present study.*

**Reviewer #1 Comments**

This is a generally well-conducted, and thoroughly analyzed study that addresses a gap in the literature on honey bee aversive conditioning -- in particular, the ability of honey bees to use members of their own species or another species as a cue to facilitate learning. Given that honey bees are eusocial, it is reasonable to predict that individual bees should be attentive to the presence of a conspecific, and should therefore be capable of using this type of stimulus as a cue; however, what the authors show is somewhat more complex. They claim that, although the stimulus organism was noticed, the presence of another organism seems to have decreased performance, especially at the beginning of a trial, when subjects often performed below chance level. This is puzzling, given that what constituted "correct" was different across conditions -- so would have to mean that sometimes, the subject got closer to the stimulus animal, sometimes, further away (sometimes one side of the box, sometimes the opposite side), but that they seem to have been drawn toward making an incorrect choice in all cases.

The biggest limitation of the present study is the large number of groups (14) and time bins (18) employed, which would necessitate a larger sample size than the one the authors used -- particularly if the expected effect size is small. I am also concerned that the authors seem to have failed to control for the multiple comparisons they conducted. Since any effects observed appear to be rather small, it is understandable that the authors would not have wished to further diminish the probability of obtaining a significant effect; however, what comes out of the various analyses is often difficult to interpret, seeming to fluctuate randomly. Personally, I would need further assurances from the authors that any patterns seen in the present study are truly indistinguishable from noise.

*The reviewer makes a number of helpful comments, and were happy to use these to improve our paper. The reviewer’s comments were regarding statistical analysis. Before addressing each point, we would like to emphasize that many aspects of traditional null-hypothesis statistical testing (NHST) are arbitrary, including the classic use of a threshold of p < 0.05. While NHST can be very useful, it is not without criticism. Some journals, like Basic and Applied Social Psychology, Epidemiology, Political Analysis have even banned reporting p-values. It is because of these criticisms that we also included raw data, several types of graphs, confidence intervals, and an assumption-free, ordinal analysis. The parameter estimates in our regression models are also free of NHST subjectivity and tell a consistent story independent of the p-values. Although we have improved our NHST analysis and the ability of readers to interpret our analysis based on the reviewer’s feedback, we believe the non NHST components of our results are still substantial.*

*With respect to sample size, 16 to 32 bees is a common number of subjects in shuttle box studies (e.g., Agarwal et al., 2011; Avalos et al., 2017; Dinges et al, 2012; 2017) and other bee conditioning experiments (Varnon, et al, 2018; Abramson, Dinges, & Wells, 2016; Junca, et al. 2014; Dobrin, & Fahrbach, 2012). This range of sample size does not appear to hinder conclusions from being made. For shuttle box research specifically, the small number of studies means it would likely be premature to estimate an expected effect size. In the discussion of our paper, we did make some comparison of our findings to other shuttle box experiments that used similar punishment-based methods. However, there were only a small number of comparable studies. Half of these studies were published after the present experiment was complete, the other half were conducted by our laboratory before the present experiment. Without an ample basis of literature to use to predict an effect size, we followed the methods that we expected would be successful. We have clarified our sample and group sizes in the subjects section of our paper, starting at line 99.*

*Junca P, Carcaud J, Moulin S, Garnery L, Sandoz J-C (2014) Genotypic Influence on Aversive Conditioning in Honeybees, Using a Novel Thermal Reinforcement Procedure. PLoS ONE 9(5): e97333. https://doi.org/10.1371/journal.pone.0097333*

*Abramson CI, Dinges CW, Wells H (2016) Operant Conditioning in Honey Bees (Apis mellifera L.): The Cap Pushing Response. PLoS ONE 11(9): e0162347. https://doi.org/10.1371/journal.pone.0162347*

*Dobrin SE, Fahrbach SE (2012) Visual Associative Learning in Restrained Honey Bees with Intact Antennae. PLoS ONE 7(6): e37666. https://doi.org/10.1371/journal.pone.0037666*

*Agarwal M, Giannoni-Guzmán, M, Morales-Matos, C, Del Valle Díaz, RA, Abramson CI, Giray, Tugril. Dopamine and octopamine influence avoidance learning of honey bees in a place preference assay. PLoS ONE. 2011; 6(9): e25371. doi: 10.1371/journal.pone.0025371*

*Avalos A, Pérez E, Vallejo L, Pérez ME, Abramson CI, Giray T. Social signals and aversive learning in honey bee drones and workers. Biol Open. 2017; 6: 41-49, doi: 10.1242/bio.021543*

*Dinges CW, Avalos A, Abramson CI, Craig DPA, Austin ZM, Varnon CA, Dal FN, Giray T, Wells H. Aversive conditioning in honey bees (Apis mellifera anatolica): A comparison of drones and workers. J Exp Biol. 2013; 216, 4124-4134. doi:10.1242/jeb.090100*

*Dinges CW, Varnon CA, Cota LD, Slykerman SC, Abramson CI. Studies of learned helplessness in honey bees (Apis mellifera L.). J Exp Psychol: Animal Learn Cogn. 2017; 43: 147-158. doi: 10.1037/xan0000133.*

*Varnon, C. A., Dinges, C. W., Black, T. E., Wells, H., & Abramson, C. I. (2018). Failure to find ethanol-induced conditioned taste aversion in honey bees (Apis mellifera L.). Alcoholism: Clinical and Experimental Research, 42(7), 1260-1270, doi: 0.1111/acer.13761.*

*With respect to the number of time bins, the reviewer makes an important point that we have also considered. Our 9-minute sessions were divided into 30-second bins for the sake of data analysis. This division was somewhat arbitrary and was designed to provide enough temporal resolution so that we could see changes in behavior across time without dividing the data into more bins than could be easily reviewed with a visual inspection. Since we initially had some continuous data, we wrote a program that could further divide or combine bins. Consider the graphs below. Here, we show CCR data for the spatial, shock on yellow, and shock on blue groups, binned into 9, 18, and 36 bins. With 9 one-minute bins, the data appears less variable that it actually is. However, with 36 bins, the variability makes inspecting the graphs a difficult task. Our 18-bin approach presents a happy medium where we can show changes across time, and also some of the variability that goes with it.*

*Like the reviewer, we initially had some concern on the appropriate number of bins for statistical analysis. Before submitting the paper, we conducted tests at different bin numbers and found there to be little impact on p-value. For the most part, repeated measures analysis are designed with in mind. However, we did make an error here, and so we are glad the reviewer offered this comment. Our initial tests used a generalized estimating equations (GEE) regression technique, and at some point, we switched to the more well-known mixed linear model (MLM) regression reported in the paper but did not retest the impact of bin number on p-values. It appears that the MLM method is far more sensitive to increases in bin number than our original GEE method. For example, in Table 2 we analyzed the effect of group and bin on CCR for the standard control groups (spatial, shock on blue, and shock on yellow).* *We reconducted this analysis at 9, 18, 36 and 72 bins using both MLM and GEE regression techniques. Below we show the parameter estimates, followed by p-values, for the Spatial x Bin interaction. While the parameter estimates remain consistent, the GEE p-values are much less sensitive to the number of bins. For this reason, we switched our entire analysis to the GEE technique. There are no substantial changes in the results; all parameter estimates are similar, if not identical, and most major findings are still significant. However, this method is much less affected by bin number and appears to provide a less biased p-value.*

| *Number of Bins* | *MLM* | *GEE* |
| --- | --- | --- |
| *9* | *2.597, 0.013* | *2.597, 0.032* |
| *18* | *0.644, 0.006* | *0.644, 0.035* |
| *36* | *0.160, 0.002* | *0.160, 0.037* |
| *72* | *0.040, 0.000* | *0.040, 0.036* |

*The reviewer also provided some useful comments regarding multiple comparisons. These comments are appreciated, and we have included additional material at the end of our regression analysis section, starting at line 272, so that readers can better interpret our pairwise comparisons. Our regression results conveniently separate all pairwise comparisons into separate tables or sub-tables where the reader can make adjustments to their threshold for a significant finding. The simplest adjustment would be a Bonferroni correction, which would divide the traditional alpha value of 0.05 by the number of pairwise comparisons being made. Consider Table 2, for example. The bottom part of the table lists pairwise comparisons between group, and pairwise comparisons between the learning parameters for group. To make a Bonferroni correction for the group effect, one would divide 0.05 by the number of groups being compared (3) to derive a new significance threshold of 0.0167. The provided p-values would be compared to the new significance threshold. The same adjustment could be done for the comparisons between the three learning rates (Group x Bin interactions).*

*It is important to point out that there are a number of corrections techniques available, with the Bonferroni correction being just one common, simple approach. The reader is free to use a Šidák, Dunnett’s, Scheffé’s or any other correction method. The choice of correction is somewhat subjective, as is the choice of a starting alpha of 0.05. We therefore encourage the reader to make their own decisions about how to interpret the p-values. What is not subjective, however, are the parameter estimates. We would like to emphasize that the parameter estimates tell a consistent story that is completely independent of the p-values.*

*Ultimately, our results show that while the performance of bees improved across the experiment (with the exception of the shock on blue group), the initial responding was reduced due to the presence of other bees or wasps as stimuli. This can be seen consistently in the graphs, ordinal analysis, and especially the parameter estimates of the regression analysis. The spatial group responds at a higher level than any other group within the first 30 seconds of the experiment. This can be seen in the graphs and in the regression analysis. Then, the spatial group shows a positive learning rate for the remainder of the session (observable in the graphs, the regression analysis, and the ordinal analysis). The experimental groups have a much lower initial response, but aside from the shock by live bee, safe by live wasp and shock by dead wasp groups, all experimental groups have a greater learning rate than the spatial group, resulting in them nearly “catching up” to the spatial group by the end of the experiment. This effect is observable in the graphs, the regression analysis, and parts are observable in the ordinal analysis. This final level of responding observed across groups appears common to the handful of similar experiments that have been conducted. The only aspects of the data that appear to fluctuate are the differences between the individual experimental groups. We would agree with the reviewer, there does not seem to be a difference between these groups, and thus we suggest that the reduced initial response effect is caused by the presence of any stimulus animal.*

Page 4, line 9, I think you meant "single" instead of signal.

*The correction has been made.*

Page 5, line 123, I am not sure I undestand. Do you mean that there are two bees simultaneoulsy in the same chamber? If so, would you not expect that to interact with whatever effect the conspecifics and interspecific animals had?

*We ran two bees simultaneously in two separate shuttle boxes. A single program records simultaneously from both apparatus, but the bees are not able to interact in anyway. We have made a clarification regarding this in the paper on line 133.*

Another question about the shuttle box: you mention that the subjects were able to physically interact with the stimulus organisms on the other side of the mesh, but did you observe them doing this? How close were the stimuli, especially the dead stimuli, to the mesh?

*We added a few notes to the paper to clarify these questions at around line 141.*

*The stimulus chambers were designed so that the animals were not constricted, but were forced to be immediately adjacent to the mesh. They were able to rotate and move to a degree but were not able to walk farther or closer away from the subject. We could not closely observe interactions during the experiment, but the subject bees do reach the mesh during normal pacing. It is very typical of bees to pace the entire length of a shuttle box in this manner. While designing the apparatus, we were able to observe more closely to confirm that the animals could interact to a degree and that antennae often protruded through the mesh.*

Page 23, line 439, I think a word is missing in the header.

*Thanks, we have made the correction.*

You explain you small and lack of effects in the context of biases established during foraging. What would you predict would happen if you used newly-emerged bees instead of experienced foragers?

*Excellent question. If one was able to acquire bees that have developed into foragers, but have not yet foraged, it is likely that the color biases would not be observed. The difference between spatial and experimental groups may still be present.*

*For newly emerged bees, the entire learning process may differ. Some of the earliest tasks that bees engage in is cleaning cells and feeding larvae. It is possible that worker bees at this stage would respond completely differently in the shuttle box task. To our knowledge, this is an unresearched area, and we added a comment regarding this to the discussion.*

Throughout: I would avoid using the term "trend" unless you are referring to an actual trend analysis.

*We removed confusing use of the word trend from our paper.*

**Reviewer #2 Comments**

In the manuscript entitled “Conspecific and intraspecific stimuli reduce aversive learning in honey bees (Apis mellifera)” (PONE-D-19-27434), the authors describe a series of experiments assessing if of honey bees can use conspecific (a bee from the same hive) or interspecific (a wasp) as cues for a place preference assay in a shuttle box. For these experiments the authors measured two variables: the “correct compartment restriction (CCR), indicating the preference of the bees, at a given time, for one or the other side of the shuttle box, and the “activity”, which indicates the number of time a bee passes from one side to the other of the shuttle box. In particular, the authors performed several experimental and control groups to test several possible effects of conspecific and interspecific cues on an aversive place preference learning task. The data are presented in a clear graphs and supplementary material figures present a graphical representation of the raw data. Along the paper, the authors proceed to a systematic description of results that makes it easy to understand the many analyses they performed. Addionally, the analysis of each experiment in three steps (visual description, regression statistics, ordinal statistics) is appreciated and allows the authors to not be constrained to only discuss the results in terms of statistical difference and p-values. Finally, the statistics used in this study are extensively explained in the material and methods section, which allows for a good understanding of the analyses.

However, some general and more specific aspects of the manuscript could be improved. On a general level, the bibliography cited in the manuscript is in several occasions rather dated or incomplete (see specific comments).

*Thanks for the suggestions. We have made all these edits and provide specific responses below.*

The graphs could be improved with colors and error bars (the error bars could represent a confidence interval already computed in the “bootstrapping” ordinal analysis) for better visualization of the experimental data.

*We added color to our graphs, with careful consideration that the graphs were still easy to interpret for individuals with color vision deficits (including some of the authors).*

*Unfortunately, including confidence intervals directly in the graph will make them very cluttered and hard to interpret. For your consideration, see the graph below. It shows data for the spatial, shock on blue, and shock on yellow groups, as seen in the original Fig 2, but adds error bars representing 95% confidence intervals (one interval calculated for each group at each bin). The variability in the data makes the confidence intervals challenging to interpret.*

*Bee learning data is often highly variable. Even in proboscis extension response conditioning experiments, variability can be high if less motivated subjects are not removed. We are not trying to hide this variability, it can be observed in our raw data, the statistics. The heatmap plots were even created specifically to allow for a visual inspection of variability. However, given the variable nature of working with bees, it does not seem useful to add confidence intervals to the line graphs.*

Also, and most importantly, the authors seem to confuse the preference for a side or another (CCR) at a given time, and the evolution of the CCR value across time (change that can be a proxy of learning the conditioning rules of the experiment). This confusion is present at several places in the manuscript and should be remedied (see specific comments).

*Yes, thank you for this point. We agree and appreciate the comment on this language. It was challenging to articulate the effect we observed without implying some other effect. We already discarded a number of phrases and terms, such as inhibit, due to potential alternative meanings. Based on the reviewer’s feedback we changed the title to: Conspecific and interspecific stimuli reduce initial performance in an aversive learning task in honey bees. We have also made small corrections throughout the paper to improve our language regarding this matter.*

On the whole, despite some corrections needed to improve or correct the manuscript, this study has enough strength to be accepted for publication.


- Throughout the manuscript, the interspecific group is sometimes mistakenly labelled intraspecific (title, introduction, etc…)

*Thanks for catching this error. We used a search function to ensure all corrections were made.*

- Line 26, authors could mention that the experiment consists in a spatial conditioning, as other studies of the same kind in honey bees usually use visual or olfactive conditioning instead.

*Excellent suggestion. We revised the abstract and introduction accordingly.*

- In this study authors conclude that learning is similar in conspecific, interspecific and spatial groups (see Table S2; Spatial X bin : Other groups X Bin). However, in the title and abstract, they state several times that social cues reduced learning. In fact, social cue only generally reduced CCR, but doesn’t impair its improvement (learning). This should absolutely be remedied.

*See our previous comment regarding our edits.*

- Line 51, citations for nestmate and non-nestmate recognition and discrimination studies in the honey bee seem a bit dated. Maybe some other studies were published more recently and could be added.

*We were able to find and add a more recent citation.*

- Line 58, the most recent review on the PER conditioning protocol is lacking from the citations and should be added (Matsumoto et al., 2012, J Neuro Methods)

*This citation has been added.*

- Line 61, a review article on visual conditioning in harnessed honey bees has been published recently and could be cited to illustrate the point (Avarguès-Weber and Mota 2016, J Physiol Paris)

*This citation has been added.*

- Line 62, recent publication by Howard and colleagues (science) have been published on numerical abilities in honey bees that would illustrate perfectly the point on quantity discrimination.

*This citation has been added.*

- Line 62, the most recent and up to date citations for the neurophysiology of memory in honey bees are (Müller 2012, Apidologie; Eisenhardt 2014, Learn & Mem).

*These citations have been added.*

- Line 65, to illustrate a broader spectrum of aversive conditioning laboratory studies, two citations could be added (Kirkerud et al., 2017; Marchal et al., 2019, Learn & Mem).

*We have the Kirkerud citation already. We added the Marchal citation.*

- Line 70, the list of the themes tackled using an aversive shuttle box kind of experimental setup could be widened citing Marchal et al., 2019 with the study of modulation of phototaxis.

*We added the Marchal citation.*

- Line 71, Marchal et al., 2019 also qualify as a reference on the role of dopamine, octopamine and mushroom bodies in aversive learning.

*We added the Marchal citation.*

- Line 72-81, the authors elaborate on the lack of studies on the modulation of aversive learning by social cues. This section could be improved by adding a statement concerning the studies that exist on shock responsiveness modulation by pheromones in honey bees, which might be interesting to discuss in the framework of this manuscript (Rossi et al., 2018, JEB).

*Thanks for the suggestion. We were unaware of this paper. We added this citation.*

- Line 123, the authors describe the setup as being able to perform the conditioning of two independent animals in parallel. How was this capacity used? Were animals of the same groups performed in parallel? Or on the opposite, were animals of different groups performed in parallel?

*This set up was used simply for the convivence of collecting data more efficiently. In previous experiments, the same apparatus was used as a master/yolk pair, but here each shuttle box ran independently. The order of conditions, location of stimuli, location of shock, and side that the bees entered the shuttle box were all randomly assigned. Each pair used the same set of conditions to reduce experimenter error. We made some revisions to the apparatus section to clarify this point.*

- The Apparatus section of the Material and Methods could benefit from a visual representation of the apparatus (that seem to be slightly different from the one used in previously published studies) to help understanding.

*Unfortunately, we no longer have access to the 3D models, but we were able to include a few images which readers may find helpful.*

- Line 148, typo: Controlled should be replaced by Controller.

*Thanks. The correction has been made.*

- Line 169-170, the description of the apparatus using the terms top half and bottom half is misleading as the terms top and bottom were previously used to describe what could be called floor and ceiling of the apparatus. This highlights again the improvement that a visual on the apparatus could ease understanding.

*We used the reviewers suggested terms of ceiling and floor and revised the apparatus description accordingly.*

- Line 170, the text seems to suggest that a side of the apparatus was always associated to yellow and the other side to blue, indicating that the sides were not balanced with respect to the color stimuli. If this is the case, it might imply a spatial bias in the experiment.

*Yes, thank you for noticing this. Shock location was counterbalanced for the entire experiment. We revised our description of the methods to be clearer.*

- Line 227, contrasting with the rest of the statistical analysis description, the part explaining the z-score of activity might be missing clarity. It is not clear with respect to what mean value the z-scores are computed. Is it to the mean of all groups of a same experiment at a given bin? Is it to the mean of all bins of a single group?

*Excellent point. The z scores were calculated by reference to the mean of all z scores in the entire experiment, including all groups and bins. We clarified this in the paper.*

- Line 296, the authors state that “the bias appears to dissipate” because the CCR is improving across bins. However, the increase is probably due to learning, while the bias is still present.

*We have revised this statement to be clearer and now state “… this bias is less apparent later in the experiment as CCR improves above the chance level, likely due to learning.”*

- Line 314, the low CCR bees in the color discrimination groups show low activity too, which means that the bees stayed in the shocked compartment (keeping receiving shocks), which might indicate that those bees were not feeling the shock, or behaviorally impaired. This possibility could be discussed.

*Yes, both low and high CCR were often associated with low activity levels. Generally, we observed low activity was associated with pacing in a smaller area, even when shock was received. We added a note regarding this to the results.*

*The apparatus was designed and tested to ensure the bees could not avoid shock. The small inner space of the shuttle box, as well as the presence of both a floor and ceiling shock grid ensures the bees always receive the shock. Additionally, the level of the shock is set to a point where behavioral responses to shock are obvious and consistent, without physically impairing the bees or causing stinging or vomiting that may lead to increased conductivity and additional shock being received. This general method is a fine balance that we have refined across a number of experiments. We added a note on this to the apparatus section.*

*We also discarded any bees that were completely immobile once placed in the apparatus. We added a note regarding this to the subjects section.*

- Line 367, “effect” could be replaced by “activity”.

*We tried to follow the reviewer’s suggestion, without also ending the sentence and starting the following sentence with “activity.” We now state “In this case, the activity level of the bee and wasp baseline is an intermediate of the other groups.”*

- Line 368, “illustrate” should be replaced by “illustrating”.

*The correction has been made.*

- Line 382, the study does not seem to specifically test a general preference (all bins pulled) towards stimulus bee or stimulus wasp which could be interesting to assess (as a post hoc analysis for example).

*We added a post hoc single sample t test and again found a very small, nonsignificant preference towards being near a stimulus bee and away from a stimulus wasp.*

- Line 435, “High” should be added before “CCR”.

*Thanks. The correction has been made.*

- Line 448, as the conspecific and interspecific groups are compared to the spatial group, it might improve understanding of the data to make the spatial group appear on all conspecific and interspecific graphs for visual comparison.

*We followed the reviewer’s suggestion.*

- Line 488, what does the expression “initially reduces learning” mean? Learning being a change in behavior across time, the term initial learning does not seem to fit. This could be replaced by “reduces the overall CCR level”.

*As with the earlier comment on this general topic, we agree and appreciate the suggestion. It has been incorporated into the paper.*

- Line 489, the authors state that the spatial group reached a final level of 66%. However, to be precise, this value is extracted from the model that fitted the data, not the measured behavior of the animals. The final CCR level that the spatial group reached is slightly lower and should be the one reported here.

*The final data point for the spatial group is 61%. We followed the reviewer’s suggestion and also included this in the discussion.*

- Line 498, stating that the study has been performed with the same experimental set up and protocol would strengthen the argument.

*The suggested edits have been made.*

- line 503, the authors argue that the low CCR (compared to the spatial group) of bees in the shock on blue and shock on yellow groups is likely due to prior experience of bees on colored flower. However, if both groups were performed in parallel, and given the fact that they were exposed to blue and yellow cues at the same time in both groups, a bias would logically only make them learn better in one case or in the other (with a constant bias towards one of the colors). However, the experiment shows a lower CCR of both those groups compared to the spatial group. Hence, the bias resulting from prior experience is not likely to explain the low CCR. However, authors also demonstrate that the spatial and shock on yellow groups learn equally (see fig 2: Spatial X bin : Shock on yellow X bin) while shock on blue group do not learn. This might be due to a bias resulting from more experience feeding on blue than yellow flowers, hence difficulties to associate the blue color to an aversive stimulus.

*Yes, we agree with this assessment, but do not think any edits are needed. Our final statement in this paragraph closely matches that of the reviewer “our bees also likely had more experience feeding on blue than on yellow flowers.” Given the prevalence and potential complexity of color biases in bee research, work specifically focused on this topic is clearly needed.*

- Line 523, the statement about sensitization and habituation is not clear.

*We suggest that the typical shuttling behavior of bees may be somewhat incompatible with good performance in this type of task. When a stimulus animal is present, this may facilitate more shuttling – not necessarily directed toward or away from a stimulus animal. As the subject habituates to the stimulus animal, the overall activity decreases, and behavior can be altered more by the aversive contingencies.*

*We revised this section of the discussion to further clarify this point.*

- Line 526, are paper wasps known antagonists of honey bees? If not, there might not be any reason to expect an antagonistic behavior with paper wasps.

*Yes, Polistes and especially Vespula wasps are known to compete with honey bees, and we have also observed these often-aggressive interactions at our feeders. In our observations, the bees typically give the wasps space, but guard bees are also known to aggressively defend the hive from invading wasps. We added an additional citation and clarification.*

- Line 526, syntax.

*This section has been rewritten as part of one of the reviewer’s previous comments.*

- Line 540, the authors describe an alternative punishment method without much precision. This could be elaborated further for better understanding.

*We have clarified the negative reinforcement vs positive punishment procedures often used in shuttle box work.*

- Line 565, “collecting” should be replaced by “collected”.

*Thanks. The correction has been made.*

- Line 576, authors should replace “overall learning” for “preference level”. The learning performances have been demonstrated to be similar.

*We revised this to “… reduces initial performance” based on several of the reviewer’s earlier comments.*

- Line 587, as the intended negative controls (dead animal stimuli, compared to live ones) did show the same reduction of overall CCR compared to spatial group, this study would have benefitted from the use of additional controls to isolate the parameter that induces the reduction of CCR. For example, some studies use dummies to control for the possible chemical detection of the stimulus animal. In absence of such control (demonstrating no effect on behavior compared to the spatial group), one could argue that the measured effect (decrease in CCR levels) could be due to unintended experimental bias. This could be discussed somehow.

*Yes, the use of fatty acids that aid in hive mate recognition that we mentioned would be one such future investigation that could help identify the cause of the effect we observed. This could be combined with dummy controls and other methods as the reviewer suggested, and we added a comment to the discussion to expand on this point.*

- Line 591, the earlier version of the visual APIS setup (Kirkerud et al., 2017) already used multiple IR beams to assess position and velocity of bees in the apparatus.

*Yes, this is an excellent apparatus. We initially included only a single example to be concise, but have now added this citation as well.*

- Line 592, Marchal and colleagues, 2019 also used a video analysis method for precise honey bee tracking inside a visual aversive shuttle box.

*We added this citation as well.*

- Line 592-594, the idea developed by the authors in this sentence would benefit from a more explicit statement in order to be understandable without having to read the cited paper.

*Good suggestion. We added an example to clarify one of the principles, but we are not able to elaborate on all the principles. Thompson and Spencer 1966 and Groves and Thompson 1970 papers are considered major foundation papers on principles of non-associative learning. We suspect many readers will be familiar with them. The Raklin et al paper revisits these classics 40 years later and finds the principles to still be very accurate and useful.*
