# Supplementary material for: Conspecific and interspecific stimuli reduce initial performance in an aversive learning task in honey bees (Apis mellifera)
Source: PLoS One. 2020 Feb 25;15(2):e0228161. doi: 10.1371/journal.pone.0228161 (PMC7041878; doi:10.1371/journal.pone.0228161)
Supplement: Attachment — Submitted filename: Response to Reviewers.docx [file pone.0228161.s026.docx]

**Response to Editor and Reviewer Comments**

*We appreciate the efforts of the reviewers, especially in regard to speed. We have revised the manuscript and we respond to specific comments below, in italics.*

**Reviewer #1 Comments**

I should not be up to the reader to decide if and how to correct for multiple comparisons. However, this is a relatively minor quibble.

*While the reviewer is absolutely correct in suggesting we include a correction for multiple comparisons, the selection of technique is somewhat a matter of opinion. We are aware of nearly a dozen techniques to make corrections for multiple comparisons. For this reason, we explain the simple Bonferroni procedure for the reader, while also stating that the reader is free to select other methods. The Bonferroni procedure we provided is even criticized for being too conservative; other procedures, such as the Šidák correction would actually increase the likeliness we would find a p-value below the threshold. To be unbiased, we selected a simple, fair procedure; not necessarily the one that was mostly likely to give us significance. We believe we fairly addressed this concern in the last revision, but now we also added several “reminders” for readers on where and how the Bonferroni or other correction could be applied.*

The graphs alone, especially lacking any sort of error bar, do not tell a compelling enough story.

*Error bars are excellent additions to many graphs, especially simple bar graphs where the data can be compared side by side, but error bars can be very challenging to implement on line graphs with multiple lines. We did a quick review of 15 articles published in PLOS ONE by considering the first 5 hits for searches 3 searches on “operant conditioning,” “classical conditioning,” and “honey bee learning.” Of these 15 articles, 11 contained line graphs, and of these, only 3 used error bars on the line graphs. This is a small convenience sample, but in our experience, this is the trend for line graphs in learning/behavior research with multiple groups, and likely occurs given the difficulty of interpreting many overlapping error bars.*

*Specific to honey bee learning, you may consider:*

[*https://journals.plos.org/plosone/article?id=10.1371/journal.pone.0037666*](https://journals.plos.org/plosone/article?id=10.1371/journal.pone.0037666)

[*https://journals.plos.org/plosone/article?id=10.1371/journal.pone.0040848*](https://journals.plos.org/plosone/article?id=10.1371/journal.pone.0040848)

[*https://journals.plos.org/plosone/article?id=10.1371/journal.pone.0066295*](https://journals.plos.org/plosone/article?id=10.1371/journal.pone.0066295)

[*https://journals.plos.org/plosone/article?id=10.1371/journal.pone.0004197*](https://journals.plos.org/plosone/article?id=10.1371/journal.pone.0004197)

[*https://journals.plos.org/plosone/article?id=10.1371/journal.pone.0113630*](https://journals.plos.org/plosone/article?id=10.1371/journal.pone.0113630)

*In our last revision, the other reviewer also made a similar suggestion to include error bars. After reviewing a sample we included with error bars, they appear to agree that including error bars was not useful for this experiment. In our paper, we provided standard errors, confidence intervals, heat map plots, and raw data that can all show variability in our data in place of error bars. We have been as transparent with our data as possible, and we believe that error bars detract from visual inspection of the data in this case. That being said, we did our best to create graphs that included error bars for this revision. We accept the graphs in this form, but ultimately hope that you agree that the error bars are not especially helpful for this particular experiment.*

*We would also like note that overlap of error bars and statistical significance are independent topics; this is a common misconception. See for example: Payton, Greenstone & Schenker’s 2003 paper titled “Overlapping confidence intervals or standard error intervals: What do they mean in terms of statistical significance?”*

*Additionally, we do not overstate our results or try to tell a “compelling story”; we simply described what was observed. Our work here does not exist to support or refute any particular theory, rather it is research regarding a new topic (social stimuli in aversive learning in honey bees) that is also part of a relatively less researched area (aversive learning in honey bees).*

Outside the difference between the spatial group and all other groups, and the general learning curve, I cannot see patterns clear enough in these data to warrant drawing definitive conclusions or dispensing with p-values, arbitrary as they may be. And the statistics you have provided do not strongly support your interpretation, either, in my opinion.

*Yes, we agree, the spatial group is distinct, and the other groups are not. Here, the reviewer’s interpretation appears identical to our own. The main conclusion is that the spatial group is distinct. We do not try to make distinctions between the various conspecific and interspecific groups – the data are similar. Additionally, we did not dispense or reject p-values. Although criticisms of null hypothesis testing can be seen as early as the 1960 (for some early discussion, see Krantz’s “The Null Hypothesis Testing Controversy in Psychology”), we still fully conduct traditional statistical analysis. We provide this analysis, including p-values and multiple comparison corrections. Given the substantial criticism of null hypothesis statistics, we also provide the reader with alternatives to supplement traditional analysis, but by no means discard p-values altogether. All our data, graphs, and analysis point in the same direction. We have been very transparent about this.*

In my previous review, I said: "They claim that, although the stimulus organism was noticed, the presence of another organism seems to have decreased performance, especially at the beginning of a trial, when subjects often performed below chance level. This is puzzling, given that what constituted "correct" was different across conditions -- so would have to mean that sometimes, the subject got closer to the stimulus animal, sometimes, further away (sometimes one side of the box, sometimes the opposite side), but that they seem to have been drawn toward making an incorrect choice in all cases." I was unable to find a place in the text or the response that dealt with this issue.

*We apologize for not directly addressing this earlier. We did not address this in our last response as it seemed to be part of the reviewer’s overall summary, not a criticism. The reviewer’s statement essentially describes a major finding of our experiment, and we felt this was clear. In our earlier revision, reviewer #2 offered some comments on this point, and we added additional clarification based on reviewer #2’s comments. This general topic is addressed in the section titled “Reduced Performance in the Conspecific and Interspecific Groups.”*

*In essence, it is not that the bees are drawn toward the incorrect chamber (aside from color biases), they simply pace more and make more errors when any stimulus animal is present. We know from previous work that rapid pacing is common in this type of procedure, and that what bees typically learn (as in the spatial group) is to restrict their pacing to one area. In our study, the groups with the highest activity scores (breaking, but not necessarily crossing, the infrared beam in the center) also have the lowest CCR scores (pacing on the correct side). It appears that the presence of stimulus animals causes more overall activity, which inhibits the ability of the bees to restrict their pacing to just one area. This initially puts the subject’s correct response at nearly chance or slightly below as chance, as the act of being shocked can slow movement and keep subjects in the incorrect area longer. As subjects habituate to the animal stimuli, it impacts their overall activity less, and subjects are then able to restrict their pacing to a small area, as they are almost immediately in the spatial group. We added a few lines and rephrased a few statements to further clarify this point in the “Reduced Performance in the Conspecific and Interspecific Groups” of the discussion.*

*While some of these results may be considered unusual by mammalian standards, there is accumulating evidence that the pacing behavior of bees in shuttle boxes is a very important aspect to consider. For this reason, in the final section of our discussion we suggest not only good temporal resolution (as in our experiment), but also good spatial resolution as in Plath et al. 2017 is an important methodological consideration for future shuttle box research.*

**Reviewer #2 Comments**
The revised version of the manuscript is, in my opinion, totally satisfactory. The authors addressed all major and minor comments and suggestions from both reviewers in a comprehensive and positive manner that led to the improvement of the manuscript.

*We appreciate the reviewer’s efforts and considerations.*
